# A comparative evaluation of salivary and serum procalcitonin to identify infants with serious bacterial infections

**DOI:** 10.1007/s00431-026-06763-3

**Published:** 2026-01-20

**Authors:** Sercan Çınarlı, Ali Yurtseven, Caner Turan, Elif Azarsız, Timur Köse, Eylem Ulaş Saz

**Affiliations:** 1 Department of Pediatrics, Faculty of Medicine, Ege University, 35100 Bornova, Izmir, Turkey; 2 Department of Clinical Biochemistry, Faculty of Medicine, Ege University, Izmir, Turkey; 3 Department of Biostatistics, Faculty of Medicine, Ege University, Izmir, Turkey

**Keywords:** Procalcitonin, Saliva, Serious bacterial infection, Infant

## Abstract

Differentiating serious bacterial infections (SBIs) from viral illnesses in infants remains challenging. Although serum procalcitonin (PCT) is a well-established biomarker, its measurement requires invasive blood sampling. This study investigated the correlation between salivary and serum PCT and evaluated the diagnostic accuracy of salivary PCT for identifying SBIs in infants under 1 year of age. This prospective observational study included 160 infants under one 1 year of age presenting to a pediatric emergency department with suspected SBI. Paired serum and saliva samples were collected. Salivary PCT and serum PCT levels were measured. Serum C-reactive protein (CRP), a routinely used inflammatory marker in pediatric emergency practice, was included as a comparator biomarker. Patients were classified into SBI and viral infection groups based on final diagnoses. The diagnostic accuracy of the biomarkers was assessed and compared using receiver operating characteristic (ROC) curve analysis.Of the 160 infants (median age 8 months; 63% male), 11.3% (*n* = 18) were diagnosed with SBI and 88.7% (*n* = 142) with viral infections. Median salivary PCT levels were markedly higher in the SBI group than in the viral infection group (69.3 pg/mL vs. <0.01 pg/mL;* p* < 0.001). The area under the curve (AUC) for diagnosing SBI was 0.92 for salivary PCT, 0.96 for serum PCT, and 0.88 for serum CRP. At a cutoff value of 31.3 pg/mL, salivary PCT demonstrated a sensitivity of 89% and a specificity of 92.3%, with a negative predictive value (NPV) of 98.7%. A weak but statistically significant correlation was found between serum and salivary PCT levels (r = 0.250; *p* = 0.001). *Conclusion: *Salivary PCT shows strong correlation with serum PCT and demonstrates high diagnostic accuracy as a non-invasive biomarker for identifying SBIs in infants. Its performance approaches that of serum PCT and exceeds that of serum CRP, highlighting its potential clinical value in reducing the need for invasive blood sampling.

**Table:** 

**What is Known:** •* Serum procalcitonin (PCT) is a well-established marker for detecting serious bacterial infections (SBI) in infants, surpassing CRP, though it requires invasive sampling.*	
**What is New:** •* Salivary PCT demonstrates comparable diagnostic performance to serum PCT and exceeds CRP, highlighting its potential as a non-invasive alternative for SBI assessment in infants.*

**What is Known:**

•* Serum procalcitonin (PCT) is a well-established marker for detecting serious bacterial infections (SBI) in infants, surpassing CRP, though it requires invasive sampling.*

**What is New:**

•* Salivary PCT demonstrates comparable diagnostic performance to serum PCT and exceeds CRP, highlighting its potential as a non-invasive alternative for SBI assessment in infants.*

## Introduction

Serious bacterial infections (SBIs), broadly defined in pediatric practice as clinically significant bacterial infections, including urinary tract infection (UTI), bacteremia, bacterial meningitis, sepsis, and, in some contexts, bacterial pneumonia, represent a major cause of morbidity and mortality in children, particularly in infants under 1 year of age [1–3]. The diagnostic process in these young patients is often complicated by non-specific clinical presentations that overlap considerably with common, self-limiting viral illnesses [4]. This uncertainty frequently necessitates laboratory testing to differentiate between bacterial and viral etiologies and to guide appropriate management, particularly regarding the initiation of antibiotics [5].

Among the established inflammatory biomarkers, procalcitonin (PCT) has emerged as a cornerstone in the diagnosis of SBIs [6]. Numerous studies have confirmed its superiority over C-reactive protein (CRP) and other markers, citing its rapid increase following a bacterial stimulus and its relative specificity, as its levels are not significantly elevated in most viral infections or non-infectious inflammatory states [5–7]. This has led to its widespread use in risk-stratifying febrile infants and guiding antibiotic stewardship [7].

However, for serum-based biomarkers such as PCT, the requirement for blood sampling represents a significant disadvantage. This procedure is particularly challenging and distressing in infants due to small and difficult-to-access veins, often leading to multiple painful attempts [8]. The associated pain, fear, and anxiety may not only have lasting adverse psychological effects on the child but also cause distress for parents [9]. These practical and ethical concerns have driven the search for reliable, non-invasive diagnostic alternatives.

Saliva has gained considerable attention as a diagnostic fluid because of its ease of collection, low cost, and non-invasive nature [10]. It contains a wide range of biomolecules, including proteins, hormones, and nucleic acids, which can reflect systemic physiological and pathological states [10]. In the context of infectious diseases, several studies have successfully measured inflammatory markers such as PCT, CRP, and various cytokines in saliva, demonstrating significant correlations with systemic levels and clinical status [11–13].

Despite these advances, the utility of salivary PCT for diagnosing SBI in infants remains largely unexplored. Although a few studies have investigated salivary PCT in specific settings such as neonatal sepsis or pediatric pneumonia, a comprehensive evaluation in a broader cohort of infants with suspected SBIs is still lacking [12–14].

The primary aim of this study was to evaluate the relationship between salivary and serum PCT levels in infants under 1 year of age with a suspected SBI and to determine the diagnostic accuracy of salivary PCT for this indication. An additional exploratory objective was to identify a preliminary optimal cutoff value for salivary PCT. Specifically, the study addresses two key research questions: (1) Is there a statistically significant correlation between salivary PCT and serum PCT levels in infants with suspected SBI? (2) How accurately do salivary PCT levels discriminate between infants with and without SBI? The null hypothesis (H₀) states that there is no significant correlation between salivary and serum PCT levels in infants with suspected SBI (*ρ* = 0). The alternative hypotheses (H₁) are that there is a significant positive correlation between salivary and serum PCT levels in this patient group (*ρ* > 0), and salivary PCT levels are significantly higher in infants with SBI compared to those without, supporting its role as a reliable non-invasive diagnostic marker. In addition, serum PCT and CRP were analyzed as comparator biomarkers to contextualize the diagnostic accuracy of salivary PCT against established serum-based inflammatory markers routinely used in clinical practice.

## Methods

### Study design and population

This prospective study was conducted at the Pediatric Emergency Department of Ege University Faculty of Medicine, a tertiary care university hospital in Izmir, Turkey. The study protocol was approved by the Ege University Medical Research Ethics Committee on April 27, 2023 (Decision No: 23–4.1 T/23) and was registered at ClinicalTrials.gov (NCT06354205). The research was conducted in strict accordance with the principles of the Declaration of Helsinki and Good Clinical Practice guidelines.

The study population consisted of infants aged 0 to 12 months who presented to the pediatric emergency department between January 2025 and June 2025 with clinical suspicion of SBI. Before any study-related procedures, written informed consent was obtained from all participants’ legal guardians.

Infants under 12 months of age were eligible for inclusion if they were evaluated in the pediatric emergency department with a clinical suspicion of SBI. In our pediatric emergency department, serum procalcitonin testing is routinely requested as part of the standard diagnostic workup for infants evaluated for suspected SBI; however, the decision to initiate diagnostic testing ultimately rests with the attending physician as part of routine clinical care. Inclusion in the final analysis required the availability of paired serum and salivary samples. Clinical suspicion of SBI was defined as the treating clinician’s decision to initiate a diagnostic evaluation for possible bacterial infection, including ordering laboratory investigations such as serum procalcitonin, C-reactive protein, blood cultures, urinalysis and/or urine culture, cerebrospinal fluid analysis, stool analysis, or joint fluid analysis, and/or radiological investigations when clinically indicated. This decision was based on the overall clinical assessment, incorporating presenting symptoms, physical examination findings, vital signs, and clinical judgment at the time of presentation. Exclusion criteria were: age greater than 12 months; presence of a known chronic inflammatory, autoimmune, or malignant disease that could independently affect biomarker levels; use of systemic antibiotics, immunomodulators, or immunosuppressive drugs within thepreceding 7 days; and a clinical diagnosis of gingivostomatitis or other significant oral inflammation.

Patients were classified into SBI or viral infection groups based on their final discharge diagnoses, which were determined independently of the research team using a comprehensive review of clinical, laboratory, and radiological findings. SBIs included meningitis, community-acquired pneumonia (CAP), bacteremia, UTI, septic arthritis, and invasive bacterial acute gastroenteritis (AGE) [2, 3, 15]. Diagnostic criteria were as follows:

- Meningitis: Identification of a bacterial pathogen in cerebrospinal fluid (CSF) by culture or polymerase chain reaction (PCR), and/or, in the absence of pathogen isolation, CSF findings consistent with bacterial infection, including elevated leukocyte count with neutrophil predominance, decreased glucose concentration, and elevated protein levels.
- Community-acquired pneumonia: A compatible clinical presentation (e.g., fever, tachypnea, respiratory distress, or abnormal auscultatory findings) in combination with laboratory markers suggestive of bacterial infection and radiographic evidence of pulmonary infiltrates or alveolar consolidation on chest X-ray, as verified by a radiologist.
- Bacteremia: Isolation of a pathogenic bacterium from at least one blood culture obtained under sterile conditions. Organisms considered members of normal skin flora—including coagulase-negative Staphylococcus (e.g., Staphylococcus epidermidis), Corynebacterium spp., Bacillus spp. (non-anthracis), Micrococcus spp., and Cutibacterium (Propionibacterium) acnes—were regarded as contaminants. For these organisms, at least two separate blood cultures with concordant growth were required to classify the finding as true bacteremia.
- Urinary tract infection: Defined in accordance with established pediatric guidelines as significant bacteriuria, demonstrated by growth of a single uropathogen at ≥ 50,000 colony-forming units (CFU)/mL from a urine specimen obtained by sterile transurethral catheterization, in association with clinical signs of infection [16]. In infants under 1 year of age, clinical features included fever or nonspecific symptoms such as irritability, poor feeding, vomiting, or lethargy. Quantitative urine culture was used as the reference standard, and urinalysis findings (e.g., pyuria and/or bacteriuria) were used as supportive evidence.
- Septic arthritis: Diagnosed by isolation or identification of a bacterial pathogen from synovial fluid using culture, or PCR in the presence of compatible clinical features, including fever, monoarticular pain, joint swelling, and limited range of motion. Diagnosis was also considered confirmed when typical bacterial pathogens were isolated from blood cultures in patients with compatible clinical features and elevated synovial fluid white blood cell counts, even if synovial fluid cultures were negative. Elevated synovial fluid white blood cell counts were defined as > 50,000 cells/µL with a predominance (> 90%) of polymorphonuclear leukocytes [17].
- Invasive bacterial acute gastroenteritis: Diagnosed by isolation of an enteric bacterial pathogen from stool culture in infants presenting with diarrhea accompanied by systemic symptoms. Although stool cultures are not routinely indicated in all cases of acute diarrhea, they were obtained in patients with severe illness, immunocompromising conditions, or comorbidities associated with increased risk of complications.

The viral infection group included patients who did not meet SBI criteria and had diagnoses such as bronchiolitis, viral upper respiratory tract infection (URTI), or viral gastroenteritis, often supported by positive viral panel results.

### Data and sample collection procedures

Upon enrollment, demographic and clinical data were recorded using a standardized case report form. Paired venous blood and unstimulated saliva samples were collected from each participant. Saliva was collected at least 2 h after feeding by gently aspirating pooled saliva from the buccal and/or sublingual space using a sterile, needleless 1 mL syringe over approximately 2 min. After collection, samples were immediately refrigerated at 4 °C and delivered to the laboratory, where they remained at this temperature for less than 1 h before being stored at − 80 °C. For patients with clinical signs of respiratory infection (e.g., bronchiolitis, pneumonia, febril URTI), a nasopharyngeal swab was also obtained for a respiratory viral panel.

### Laboratory analysis

Serum samples were centrifuged at 5000 rpm for 10 min and analyzed for PCT and CRP on the same day using routine automated immunoturbidimetric or chemiluminescent immunoassays. Saliva samples were centrifuged at 4000 × g for 10 min at 4 °C, and the supernatant was stored at − 80 °C until analysis. Salivary PCT was quantified using a high-sensitivity human PCT micro-ELISA sandwich kit (Bioassay Technology Laboratory, Shanghai, China), with a detection range of 31.25–2000 pg/mL and a sensitivity of < 18.75 pg/mL. Nasopharyngeal swabs were analyzed using a multiplex real-time RT-PCR panel.

### Statistical analysis

Data were analyzed using IBM SPSS Statistics, Version 25.0. The number of patients to be included in the study was determined based on a power analysis conducted to statistically evaluate the correlation between serum and salivary procalcitonin levels. According to the power analysis, detecting a difference between correlation coefficients of 0.25 (minimal correlation) and 0.75 (strong correlation) with 90% statistical power at a 0.05 significance level required 121 participants. To account for potential data loss, the sample size was increased, and a total of 160 patients were enrolled in the study. As all numerical variables were not normally distributed, continuous variables were presented as median and interquartile range (IQR) and compared using the Mann–Whitney *U* test. Categorical variables were compared using the Chi-square or Fisher’s Exact test. Correlation was assessed with Spearman’s rank correlation coefficient. Receiver operating characteristic (ROC) curve analysis was performed primarily to evaluate the diagnostic accuracy of salivary PCT and to determine the area under the curve (AUC). ROC analyses for serum procalcitonin and C-reactive protein were included as contextual comparators to established serum biomarkers and were not intended as formal head-to-head comparisons. Optimal cutoff values were explored using the Youden index to provide preliminary estimates of potential thresholds for salivary PCT. To assess the stability of the diagnostic performance metrics (sensitivity, specificity, positive predictive value, negative predictive value, and optimal cutoff value) and to calculate their 95% confidence intervals (CIs), we employed a non-parametric stratified bootstrap resampling method to account for the imbalance in the dataset [18]. All analyses were performed using R software. The ‘pROC’ package was used for ROC curve analysis [19]. The ‘boot’ package was utilized to generate 1000 bootstrap replicates using stratified sampling with replacement based on the outcome variable [20]. In each bootstrap iteration, the optimal cutoff value was recalculated, and the corresponding diagnostic metrics were extracted. The 95% CIs were derived using the percentile method. A *p*-value < 0.05 was considered statistically significant.

## Results

### Study population and baseline characteristics

A total of 210 infants were initially assessed; 160 were included in the study, while 50 were excluded due to high salivary viscosity or insufficient sample volume. The final cohort had a median age of 8 months (IQR 5–11), and 101 infants (63%) were male. Based on the final evaluation, 18 infants (11.3%) were classified into the SBI group, while 142 infants (88.7%) were assigned to the viral infection group. The demographic, clinical, and laboratory characteristics of both groups are summarized in Table 1.

**Table 1:** Comparison of demographic, clinical, and laboratory characteristics between SBI and viral infection groups

Variables	SBI group (*n* = 18)	Viral infection group (*n* = 142)	*p*-value
Age (months)	9 (5–11)	8 (2–11)	0.272
Sex (male), *n* (%)	10 (56)	91 (64)	0.605
Peak temperature (°C)	38.7 (38.0–39.5)	38.2 (38.0–40.0)	**0.033**
Serum PCT (ng/mL)	0.89 (0.06–46.9)	0.16 (0.06–18.6)	** < 0.001**
Salivary PCT (pg/mL)	69.3 (< 0.01–1669.0)	< 0.01 (< 0.01–106.3)	** < 0.001**
Serum CRP (mg/L)	42.0 (0.3–370.9)	7.5 (0.3–106.2)	** < 0.001**
WBC (× 10⁹/L)	12 (0.2–45.1)	10.8 (2.5–79)	0.407
ANC (× 10⁹/L)	6.9 (0–40.5)	5.1 (0.4–23.3)	0.103
ALC (× 10⁹/L)	2.6 (0.2–17.7)	4 (0.5–19)	**0.044**

*SBI* serious bacterial infections, *PCT* procalcitonin, *CRP* C-reactive protein, *WBC* white blood cell count,

*ANC* absolute neutrophil count, *ALC* absolute lymphocyte count. All values are presented as median (IQR)

Within the viral infection group, the most frequently diagnosed condition was acute bronchiolitis, accounting for 62 (38.8%) of all cases, followed by upper respiratory tract infection (n:45, 28.1%), viral pneumonia (n:26, 16.3%), and viral acute gastroenteritis (n:9, 5.6%).

In contrast, among the SBI group, the most common diagnosis was UTI (n:7, 4.4%), followed by CAP (n:5, 3.1%), acute bacterial meningitis (n:2, 1.3%), bacterial AGE (n:2, 1.3%), bacteremia (n:2, 1.3%), and septic arthritis (n:1, 0.6%). Among patients diagnosed with UTI, leukocyte esterase positivity on urinalysis was present in all patients (100%). Pyuria was detected in five patients (71.4%), bacteriuria in four patients (57.1%), and nitrite positivity in two patients (28.6%). Urine culture yielded *Escherichia coli* in five patients (71.4%) and *Klebsiella pneumoniae* in two patients (28.6%). Among the five patients diagnosed with CAP, all had fever ≥ 38.5 °C (100%), abnormal lung auscultation findings, and cough (100%), while respiratory distress was present in four patients (80%). In addition, all patients with pneumonia had serum PCT levels > 0.5 ng/mL and/or CRP levels > 20 mg/dL. In cases of bacterial meningitis, *Streptococcus pneumoniae* was isolated in one patient (50%), and *Neisseria meningitidis* was isolated in the other patient (50%). Invasive bacterial AGE pathogens included *Campylobacter jejuni* (*n* = 1) and *Salmonella enteritidis* (*n* = 1). Bacteremia cases involved *Streptococcus pneumoniae* (*n* = 1) and *Group B Streptococcus* (*n* = 1). In the patient diagnosed with septic arthritis, *Staphylococcus aureus* was identified.

### Hypothesis 1: Correlation between salivary and serum procalcitonin

**Fig. 1:**
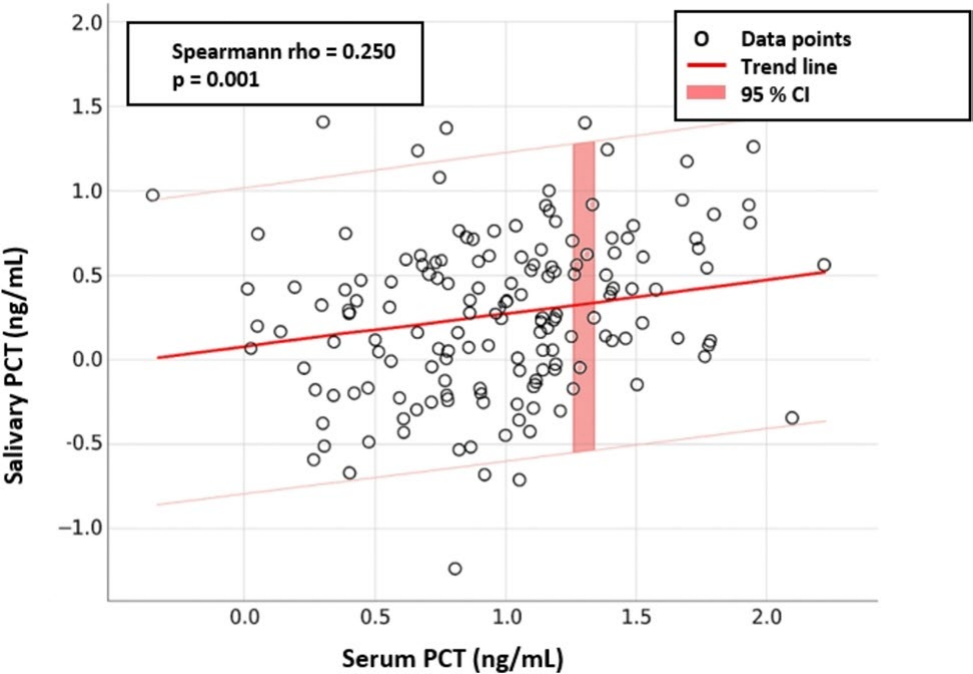
Correlation between serum and salivary procalcitonin

To test the primary hypothesis, a Spearman’s rank correlation analysis was performed. The analysis revealed a weak, but statistically significant, positive correlation between paired salivary and serum PCT concentrations across all 160 participants (rho = 0.250, *p* = 0.001) (Fig. 1). This finding leads to the rejection of the null hypothesis (H₀) that no correlation exists. However, the result provides only modest support for the alternative hypothesis (H₁), indicating that while a relationship exists, salivary PCT is not a direct linear proxy for serum PCT.

### Hypothesis 2: Diagnostic accuracy of salivary procalcitonin for SBI

To test the second hypothesis, we first compared biomarker levels between the groups. The median salivary PCT concentration was significantly higher in the SBI group compared to the viral infection group (69.3 pg/mL vs. < 0.01 pg/mL, respectively; *p* < 0.001) (Table 1). This result strongly supports the hypothesis that salivary PCT levels are elevated in the presence of SBI.

**Fig. 2:**
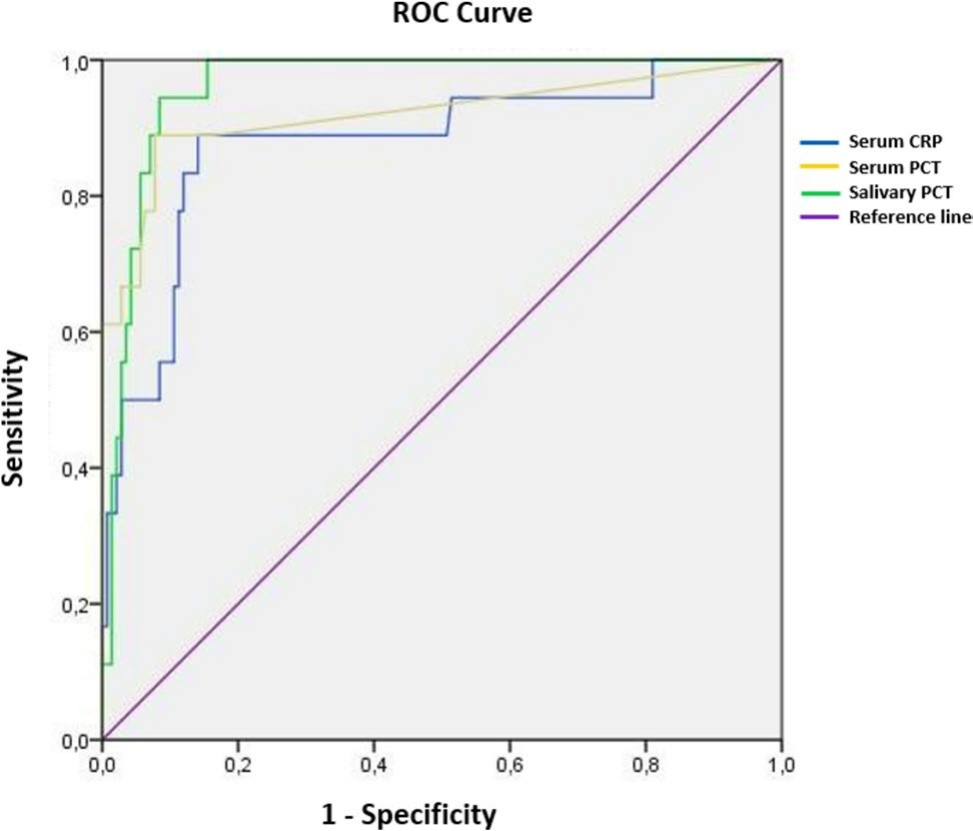
ROC curves of serum PCT, serum CRP, and salivary PCT

Receiver operating characteristic (ROC) curve analysis demonstrated that all three biomarkers had high diagnostic accuracy, which was further supported by internal validation using stratified bootstrap resampling with bias-corrected and accelerated (BCa) 95% confidence intervals. Serum CRP showed good discriminatory ability with an AUC of 0.88 (95% CI: 0.77–0.96), while serum PCT provided excellent performance with the highest AUC of 0.96 (95% CI: 0.92–0.99). Importantly, salivary PCT also exhibited excellent diagnostic accuracy, with an AUC of 0.92 (95% CI: 0.82–0.99), comparable to the AUCs observed for established serum-based biomarkers (Fig. 2). Although slightly lower than serum PCT, the diagnostic performance of salivary PCT was sufficiently high to indicate its potential as a reliable diagnostic biomarker.

At the optimal cutoff values determined by the Youden index, all three biomarkers demonstrated strong diagnostic accuracy. Serum CRP at 41.2 mg/L showed a sensitivity of 89% and specificity of 86%, with a positive predictive value (PPV) of 44.4% and a negative predictive value (NPV) of 98.7%. Serum PCT at 1.03 ng/mL provided the best overall performance, yielding a sensitivity of 94.4% and specificity of 91.5%, with a PPV of 57.1% and the highest NPV of 99.5%. Importantly, salivary PCT at 31.3 pg/mL achieved a sensitivity of 89% and specificity of 92.3%, with a PPV of 60.0% and an NPV of 98.7%. To assess the stability of diagnostic performance estimates, bootstrap resampling with 1000 iterations was performed for cutoff-based indices. Bias-corrected and accelerated (BCa) 95% confidence intervals were calculated for sensitivity, specificity, PPV, and NPV. For salivary PCT, sensitivity was 89% (95% CI: 71.4–100.0), specificity was 92.3% (95% CI: 87.9–97.0), and NPV was 98.5% (95% CI: 96.2–100.0). In contrast, PPV was 59.3% (95% CI: 41.7–85.7), with wider confidence intervals reflecting the low prevalence of SBI in the study cohort. These findings indicate that, while Youden-derived cutoff values should be interpreted as exploratory, the high sensitivity and NPV of salivary PCT remain robust across bootstrap resampling.

The detailed diagnostic performance is presented in Table 2. These results highlight that, despite being a non-invasive biomarker, salivary PCT demonstrated diagnostic performance comparable to serum-based markers, underscoring its potential value in clinical practice. However, given the limited number of SBI-positive cases and the heterogeneous spectrum of bacterial infections, these cutoff values should be interpreted as exploratory and hypothesis-generating rather than definitive.

**Table 2:** Diagnostic performance of serum and salivary biomarkers at exploratory optimal cutoff values and internal validation using stratified bootstrapping (95% CI)

Biomarker	Cutoff value (BCa 95% CI)	Sensitivity (%) (BCa 95% CI)	Specificity (%) (BCa 95% CI)	PPV (%) (BCa 95% CI)	NPV (%) (BCa 95% CI)
Serum CRP	41.2 mg/L (39.6–49.4)	89 (71.4–100)	86 (80.6–93.1)	44.4 (29.6–65.0)	98.7 (95.9–100)
Serum PCT	1.03 ng/mL (0.56–1.96)	94.4 (91.3–100)	91.5 (81.0–96.5)	57.1 (32.4–79.2)	99.5 (98.5–100)
Salivary PCT	31.3 pg/mL (27.3–54.6)	89 (71.4–100)	92.3 (87.9–97.0)	60.0 (41.7–85.7)	98.7 (96.2–100)

*PPV* positive predictive value, *NPV* negative predictive value, *PCT* procalcitonin, *CRP* C-reactive protein, *CI* confidence interval

Bootstrap resampling with 1000 iterations was used to estimate bias-corrected and accelerated (BCa) 95% CIs for sensitivity, specificity, PPV, and NPV

Due to the relatively low prevalence of serious bacterial infection (11.3%) in the study population, CIs for PPV were wider. Cutoff values derived using the Youden index should therefore be interpreted as exploratory and hypothesis-generating

## Discussion

This study investigated the potential of salivary PCT as a non-invasive biomarker for SBI in infants. Our findings demonstrate two key points: first, there is a weak but statistically significant correlation between salivary and serum PCT concentrations; second, salivary PCT displays excellent diagnostic accuracy for discriminating SBI from viral infections, with performance comparable to serum-based biomarkers.

The first alternative hypothesis (H₁) predicted a positive correlation between serum and salivary PCT. We confirmed this with a statistically significant result, allowing us to reject the null hypothesis. However, the weakness of the correlation is a critical finding. It suggests that salivary PCT levels do not simply mirror systemic concentrations and should not be used to predict the exact serum value. This contrasts with a study on pediatric pneumonia by Çelik et al., which reported a stronger correlation [13]. The discrepancy may stem from differences in our broader patient population (which included various SBI types beyond pneumonia), leading to more heterogeneity, or potential variations in sample handling and analysis. A study by Galhardo et al. in adults with sepsis also found no significant difference in salivary PCT levels compared to controls, further highlighting the complexity [21]. The weak link implies that complex physiological factors, such as local clearance mechanisms in the oral cavity or variable glandular secretion rates during systemic illness, influence salivary PCT levels independently of serum concentrations. Therefore, salivary PCT should be interpreted as a distinct biomarker rather than a direct surrogate for serum PCT.

The second alternative hypothesis (H₁), which proposed that salivary PCT could serve as a reliable diagnostic marker for SBI, was strongly supported by our findings. First, salivary PCT levels were significantly elevated in the SBI group compared with viral infections. Second, ROC curve analysis showed that salivary PCT demonstrated excellent diagnostic accuracy, with an AUC comparable to those observed for established serum-based biomarkers. This is a particularly important observation, as it indicates that a non-invasive sample may yield diagnostic information comparable to, or even better than, traditional invasive markers. Recent meta-analyses in febrile infants have reported pooled AUCs of approximately 0.81 for serum PCT and 0.80 for serum CRP, values that are consistent with our serum results and further emphasize the promising diagnostic utility of salivary PCT [22].

The clinical strength of salivary PCT, as revealed in our analysis, is its utility as a “rule-out” test. At an optimal cutoff of 31.3 pg/mL, salivary PCT achieved a sensitivity of 88.9% and specificity of 92.3%, with a negative predictive value (NPV) of 98.7%. This means that an infant with a salivary PCT level below 31.3 pg/mL has a very low probability of having an SBI. This high NPV is clinically invaluable in a pediatric emergency setting, as it could empower clinicians to confidently withhold antibiotics and avoid further invasive investigations (like lumbar punctures or catheterization) in a large subset of febrile infants, thereby reducing patient distress, healthcare costs, and antimicrobial resistance pressure. Our findings are consistent with emerging literature. For instance, Rezk et al. recently investigated children under 5 years of age with community-acquired pneumonia and reported that a salivary PCT cutoff value of 68.5 pg/mL achieved 100% sensitivity [23]. This aligns with our observation that salivary PCT can serve as a highly sensitive diagnostic tool for SBIs, although their study was limited to pneumonia, whereas our cohort included a broader spectrum of SBIs.

The strengths of our study include demonstrating that salivary PCT can be integrated as a non-invasive biomarker into the diagnostic approach for febrile infants, with its excellent negative predictive value supporting its role as a reliable “rule-out” test to reduce unnecessary antibiotic use and invasive procedures, while the ease and safety of saliva collection make it a practical, patient-friendly tool, particularly valuable in resource-limited settings and for repeated monitoring during illness.

The study has several limitations. First, its single-center design may restrict the generalizability of the findings. Second, the relatively small number of SBI-positive cases (*n* = 18) represents an important limitation and may reduce the precision of diagnostic accuracy estimates. In particular, a low disease prevalence is known to substantially influence predictive values, especially positive predictive value, which should therefore be interpreted with caution in this cohort. Additionally, the limited number of SBI cases affects the stability of certain performance metrics derived from ROC analysis. While overall diagnostic performance, as reflected by the area under the ROC curve (AUC), was internally validated using bootstrap resampling with 1000 iterations and bias-corrected and accelerated confidence intervals, cutoff values derived using the Youden index may be less stable in this context. Accordingly, Youden-derived cutoff values should be considered exploratory, as their robustness could not be formally assessed through resampling or external validation. Furthermore, enrollment based on clinical suspicion of SBI and the clinician’s decision to order serum procalcitonin may have introduced selection bias due to inter-clinician variability; however, this approach reflects real-world emergency department practice, and final diagnostic classification was based on predefined objective criteria. Although neonates were eligible for inclusion, none were ultimately enrolled, primarily due to parental reluctance to participate; therefore, the findings may not be directly generalizable to the neonatal population. Finally, a significant sample loss occurred due to difficulties in obtaining adequate saliva from infants. This is a well-documented challenge in pediatric research and represents a critical barrier to clinical implementation. The development of optimized, age-appropriate saliva collection devices will be essential to overcome this limitation and facilitate future applications.

In conclusion, our findings suggest that salivary PCT is a promising non-invasive biomarker for the risk stratification of infants with suspected SBI. Although it is not a direct surrogate for serum PCT, its high sensitivity and excellent negative predictive value make it a valuable tool for ruling out bacterial infections. A simple, pain-free saliva test could significantly improve the patient experience, reduce unnecessary invasive procedures, and support antimicrobial stewardship in pediatric emergency settings. Future large-scale, multicenter studies are needed to validate these results and to establish standardized, age-appropriate saliva collection protocols for clinical implementation.

## Data Availability

No datasets were generated or analysed during the current study.

## Abbreviations

AGE: Acute gastroenteritis
AUC: Area under the curve
CRP: C-Reactive Protein
CAP: Community-acquired pneumonia
IQR: Interquartile range
NPV: Negative predictive value
PCR: Polymerase chain reaction
PCT: Procalcitonin
PPV: Positive predictive value
ROC: Receiver operating characteristic
SBI: Serious bacterial infection
UTI: Urinary tract infection
URTI: Upper respiratory tract infection

## References

[1] Perin J, Mulick A, Yeung D et al (2022) Global, regional, and national causes of under-5 mortality in 2000-19: an updated systematic analysis with implications for the sustainable development goals. Lancet Child Adolesc Health 6:106–115 34800370 10.1016/S2352-4642(21)00311-4 PMC8786667

[2] Bernardi L, Bossù G, Dal Canto G, Giannì G, Esposito S (2024) Biomarkers for serious bacterial infections in febrile children. Biomolecules 14(1):97–110 38254697 10.3390/biom14010097 PMC10813546

[3] Mahajan P, Grzybowski M, Chen X, Kannikeswaran N, Stanley R, Singal B, Hoyle J Jr, Borgialli D, Duffy E, Kuppermann N (2014) Procalcitonin as a marker of serious bacterial infections in febrile children younger than 3 years old. Acad Emerg Med 21(2):171–179 24673673 10.1111/acem.12316

[4] Tsao YT, Tsai YH, Liao WT, Shen CJ, Shen CF, Cheng CM (2020) Differential markers of bacterial and viral infections in children for point-of-care testing. Trends Mol Med 26(12):1118–1132 33008730 10.1016/j.molmed.2020.09.004 PMC7522093

[5] Sutiman N, Khoo ZX, Ong GYK, Piragasam R, Chong SL (2022) Validation and comparison of the PECARN rule, step-by-step approach and lab-score for predicting serious and invasive bacterial infections in young febrile infants. Ann Acad Med Singap 51(10):595–604 36317570

[6] Norman-Bruce H, Umana E, Mills C et al (2024) Diagnostic test accuracy of procalcitonin and C-reactive protein for predicting invasive and serious bacterial infections in young febrile infants: a systematic review and meta-analysis. Lancet Child Adolesc Health 8(5):358–368 38499017 10.1016/S2352-4642(24)00021-X

[7] Pantell RH, Roberts KB, Adams WG et al (2021) Evaluation and management of well-appearing febrile infants 8 to 60 days old. Pediatrics 148(2):e2021052228 34281996 10.1542/peds.2021-052228

[8] Cuper NJ, De Graaff JC, Van Dijk ATH, Verdaasdonk RM, Van Der Werff DBM, Kalkman CJ (2012) Predictive factors for difficult intravenous cannulation in pediatric patients at a tertiary pediatric hospital. Paediatr Anaesth 22(3):223–229 21851476 10.1111/j.1460-9592.2011.03685.x

[9] McMurtry CM, Riddell RP, Taddio A et al (2015) Far from “just a poke”: common painful needle procedures and the development of needle fear. Clin J Pain 31(10):3–11 10.1097/AJP.0000000000000272 PMC4900413 26352920

[10] Kaczor-Urbanowicz KE, Martin Carreras-Presas C, Aro K, Tu M, Garcia-Godoy F, Wong DT (2017) Saliva diagnostics - current views and directions. Exp Biol Med (Maywood) 242(5):459–472 27903834 10.1177/1535370216681550 PMC5367650

[11] Sawhney A, Ralli M (2020) Comparison of salivary and serum c reactive protein levels in periodontitis and healthy patients using ELISA - a clinico pathological study. J Dent Res Rev 7(4):165–170

[12] Tsai CM, Tang KS, Cheng MC et al (2020) Use of saliva sample to detect C-reactive protein in children with pneumonia. Pediatr Pulmonol 55(9):2457–2462 32633868 10.1002/ppul.24947

[13] Çelik E, Kara SS, Çevik Ö (2022) The potential use of saliva as a biofluid for systemic inflammatory response monitoring in children with pneumonia. Indian J Pediatr 89(5):477–483 34595601 10.1007/s12098-021-03973-5

[14] El Taher SM, El-sayed HF, Omran AG, Abdallah MO (2023) Assessment of salivary versus serum procalcitonin level in diagnosis of late onset neonatal sepsis in preterm neonates. Suez Canal Univ Med J 26(9):0–0

[15] Lee CC, Cheng JS, Chang YJ, Chen YC, Hsin YC, Chiu CH (2022) Serious bacterial infections in young children with fever without source after discharge from emergency department: a National Health Insurance database cohort study. Pediatr Neonatol 63(5):527–534 35871150 10.1016/j.pedneo.2022.03.020

[16] SUBCOMMITTEE ON URINARY TRACT INFECTION (2016) Reaffirmation of AAP clinical practice guideline: the diagnosis and management of the initial urinary tract infection in febrile infants and young children 2-24 months of age. Pediatrics 138(6):e20163026 27940735 10.1542/peds.2016-3026

[17] Hooper M, Morones M, Rosenfeld S, Vallejo JG, Kaplan SL, McNeil JC (2025) The microbiology and clinical presentation of acute bacterial arthritis in Houston area children <5 years old in the era of molecular diagnostics. Pediatr Infect Dis J 44(8):735–741 40168616 10.1097/INF.0000000000004794

[18] Poi BP (2002) From the help desk: some bootstrapping techniques. The Stata Journal: Promoting Communications on Statistics and Stata 4(3):312–328

[19] Robin X, Turck N, Hainard A, Tiberti N, Lisacek F, Sanchez JC, Müller M (2011) pROC: an open-source package for R and S+ to analyze and compare ROC curves. BMC Bioinformatics 12:77 21414208 10.1186/1471-2105-12-77 PMC3068975

[20] Canty A, Ripley BD (2021) boot: Bootstrap R (S-Plus) Functions. R package version 1(1):3–28

[21] Galhardo LF, Ruivo GF, de Oliveira LD et al (2020) Inflammatory markers in saliva for diagnosis of sepsis of hospitalizes patients. Eur J Clin Invest 50(5):e13219 32129475 10.1111/eci.13219

[22] Milcent K, Faesch S, Gras-Le Guen C et al (2016) Use of procalcitonin assays to predict serious bacterial infection in young febrile infants. JAMA Pediatr 170(1):62–69 26595253 10.1001/jamapediatrics.2015.3210

[23] Rezk A, Bakry N, Elfiky S, Metawaa M, Ibrahim A (2025) Diagnostic and prognostic utility of salivary and serum procalcitonin, interleukin-6, and interleukin-10 in pediatric pneumonia: a prospective case-control study. Front Pediatr 13:1627451 41018059 10.3389/fped.2025.1627451 PMC12460304

